# Supplementary material for: A Critical View of the Application of the APEX Software (Aqueous Photochemistry of Environmentally-Occurring Xenobiotics) to Predict Photoreaction Kinetics in Surface Freshwaters
Source: Molecules. 2019 Dec 18;25(1):9. doi: 10.3390/molecules25010009 (PMC7017383; doi:10.3390/molecules25010009)
Supplement: Supplementary file 1 [file molecules-25-00009-s001.zip › 3.2.4_gcc-4.4.0.zip!/3.2.4_gcc-4.4.0/3.2.4_gcc-4.4.0/doc/octave/html/interpreter/Commands-For-Text.html]

Commands For Text - Untitled


Next: Commands For Completion,
Previous: Killing and Yanking,
Up: Command Line Editing


---

#### 2.4.3 Commands For Changing Text

The following commands can be used for entering characters that would
otherwise have a special meaning (e.g., <TAB>, `C-q`, etc.), or
for quickly correcting typing mistakes.

`C-q` `C-v`: Add the next character that you type to the line verbatim. This is how to insert things like `C-q` for example. `M-<TAB>`: Insert a tab character. `C-t`: Drag the character before the cursor forward over the character at the cursor, also moving the cursor forward. If the cursor is at the end of the line, then transpose the two characters before it. `M-t`: Drag the word behind the cursor past the word in front of the cursor moving the cursor over that word as well. `M-u`: Uppercase the characters following the cursor to the end of the current (or following) word, moving the cursor to the end of the word. `M-l`: Lowercase the characters following the cursor to the end of the current (or following) word, moving the cursor to the end of the word. `M-c`: Uppercase the character following the cursor (or the beginning of the next word if the cursor is between words), moving the cursor to the end of the word.
